# Articular flare-up of systemic scleroderma revealing a rare form of tuberculosis of the shoulder and extensor tendons: a case report and literature review

**DOI:** 10.1099/acmi.0.001023.v3

**Published:** 2025-10-01

**Authors:** H. Zouaki, H. Laatiris, L. Taoubane, A. Mejjad, H. Toufik, N. Elouardi, A. Bezza

**Affiliations:** 1 Rheumatology Department, Mohammed V Military Instruction Hospital, Rabat, Morocco; 2 Central Bacteriology Laboratory, Mohammed V Military Instruction Hospital, Rabat, Morocco

**Keywords:** extrapulmonary tuberculosis, systemic scleroderma, tuberculous arthritis, tuberculous tenosynovitis

## Abstract

Tuberculosis remains a major public health concern, particularly in countries where it is still endemic. Tuberculous bursitis and tenosynovitis are rare extrapulmonary manifestations, and their association with systemic autoimmune diseases such as scleroderma is seldom reported in the literature. We report the case of a 61-year-old patient with systemic scleroderma, complicated by diffuse interstitial lung disease and treated with mycophenolate mofetil, who developed tuberculous shoulder bursitis and wrist extensor tenosynovitis. The microbiological diagnosis was confirmed by ultrasound-guided aspiration of the subacromial-subdeltoid bursa, revealing the presence of *Mycobacterium tuberculosis*, detected by Ziehl–Neelsen staining, GeneXpert PCR and culture. Histological analysis of synovial tissue fragments demonstrated epithelioid granulomas with caseous necrosis, confirming the tuberculous origin.

## Data Summary

No data were produced during this research, nor are any data necessary for reproducing the work.

## Introduction

Despite ongoing efforts to ensure early detection and treatment, tuberculosis remains a major public health concern in Morocco, a country classified as having a moderate tuberculosis incidence according to international criteria [1]. Osteoarticular tuberculosis is a rare form of extrapulmonary tuberculosis, and the involvement of the shoulder and tendon sheaths, particularly in the form of tenosynovitis, is exceptionally uncommon [2,3]. When it occurs in the context of a systemic disease affecting the joints, such as scleroderma, the diagnosis becomes even more challenging. Indeed, the association between scleroderma and tuberculosis is rarely documented in the medical literature. We report a rare case of tuberculous arthritis of the shoulder associated with tuberculous tenosynovitis of the wrist in a patient receiving immunosuppressive therapy for systemic scleroderma. The insidious onset of symptoms, initially interpreted as a flare of chronic arthralgia related to the underlying disease, contributed to a delay in consultation and diagnosis. The diagnosis was ultimately confirmed by joint aspiration, which revealed purulent fluid rich in *Mycobacterium tuberculosis*.

## Case presentation

The patient was 61 years old and had received the Bacillus Calmette Guerin (BCG) vaccination in infancy. He had no previous history of exposure to tuberculosis.

Followed for 6 months for systemic scleroderma associated with interstitial lung disease (ILD), the diagnosis was established based on the following criteria: skin sclerosis, swollen fingers, reduced mouth opening, Raynaud’s phenomenon, polyarthritis affecting both large and small joints (shoulders, elbows, wrists, metacarpophalangeal joints, knees and ankles), biological inflammatory syndrome [C-reactive protein (CRP) at 34 mg l^−1^] and positive anti-Scl 70 antibodies. Additionally, the presence of New York Heart Association (NYHA) stage 2 dyspnoea, chronic dry cough and restrictive impairment on plethysmography, with confirmation of ILD on thoracic computed tomography (CT), further supported the diagnosis.

As a result, the patient was started on corticosteroid therapy at 40 mg per day, with progressive tapering until discontinuation, and mycophenolate mofetil (2 g per day) was introduced. After 3 months of treatment, there was significant improvement in respiratory function and moderate improvement in joint symptoms, although joint stiffness persisted.

Then, 3 weeks prior to his consultation, a sudden onset of afebrile monoarthritis of the right shoulder appeared, accompanied by inflammatory pain and swelling over the right wrist, with no improvement despite symptomatic treatment (paracetamol) taken by the patient as self-medication. This evolution occurred in a context of weight loss (6 kg over 3 weeks), without night sweats, cervical pain or other extra-articular manifestations, leading to his consultation at the Rheumatology Department of the Mohamed V Military Teaching Hospital and subsequent hospitalization.

Clinical examination revealed an emaciated patient with a temperature of 37 °C (after stopping paracetamol) and a painful, warm swelling of the shoulder and overlying the right wrist extensor tendons, with impossible mobilization of the two joints because of the pain. The rest of the examination was without particularity.

**Fig. 1.**
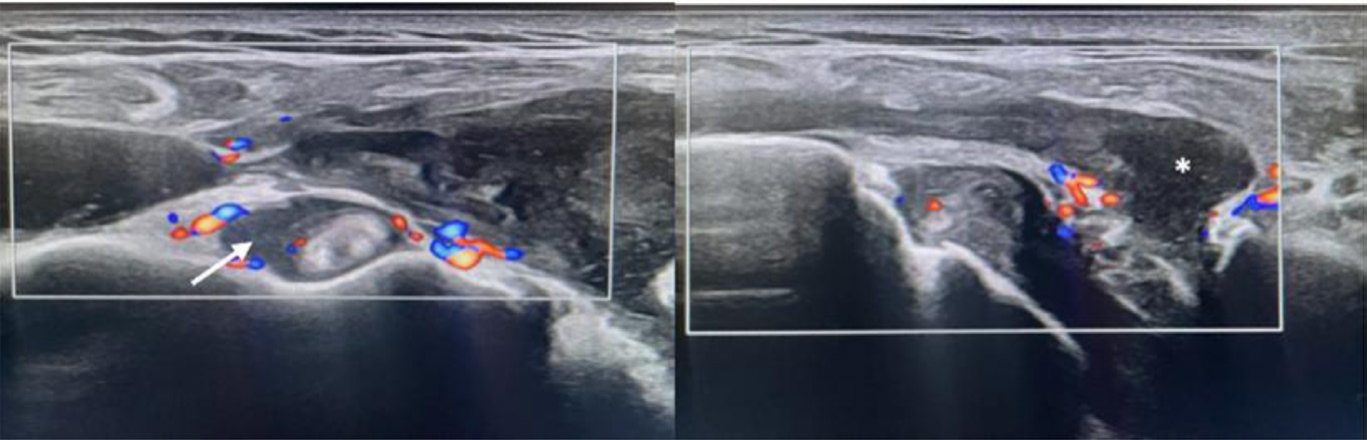
Ultrasound scan of the left shoulder showing subacromial-deltoid bursitis (asterisk) and tenosynovitis of the long biceps tendon (arrow).

**Fig. 2.**
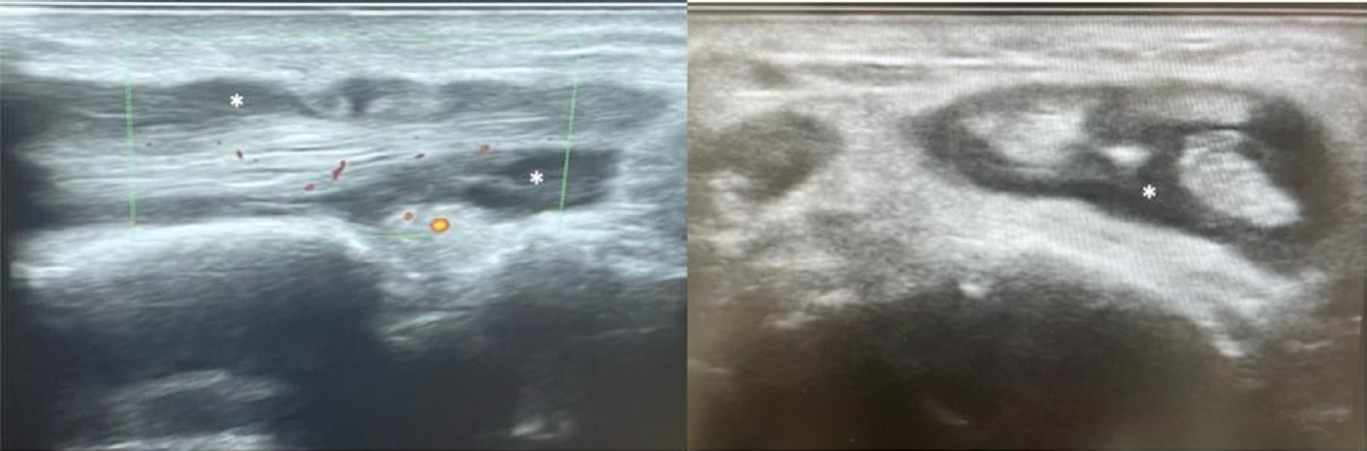
Longitudinal (left) and axial (right) ultrasound images showing tenosynovitis of the radial tendons of the right wrist (asterisk).

Given this presentation, an urgent musculoskeletal ultrasound was performed, revealing a large effusion in the right glenohumeral joint, associated with a sizable subacromial-subdeltoid bursitis (Fig. 1). Synovial sheath thickening was also noted, suggestive of tenosynovitis of the long abductor tendon of the thumb, short extensor tendon of the thumb, radial extensor tendons of the wrist and long extensor tendon of the thumb on the right (Fig. 2). An aspiration of the bursa was subsequently performed.

## Biological workup and imaging findings

**Fig. 3.**
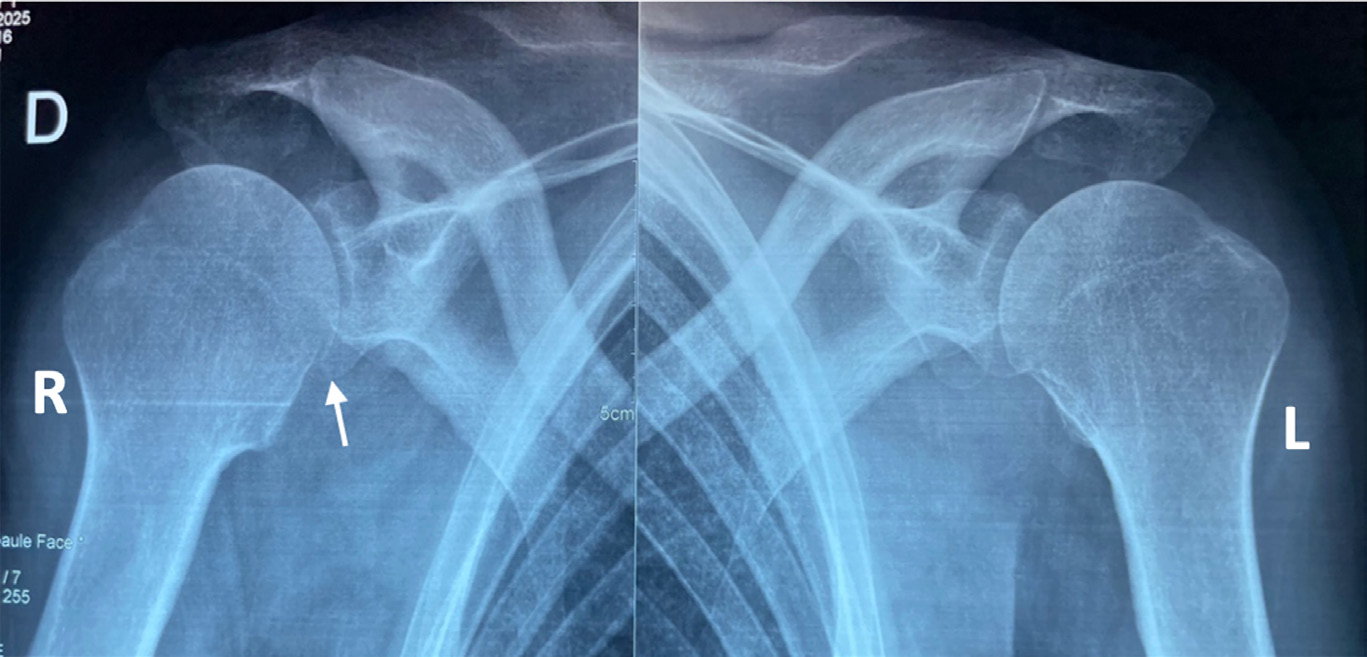
Frontal radiograph of the two shoulders showing a right glenohumeral pinch (arrow).

**Fig. 4.**
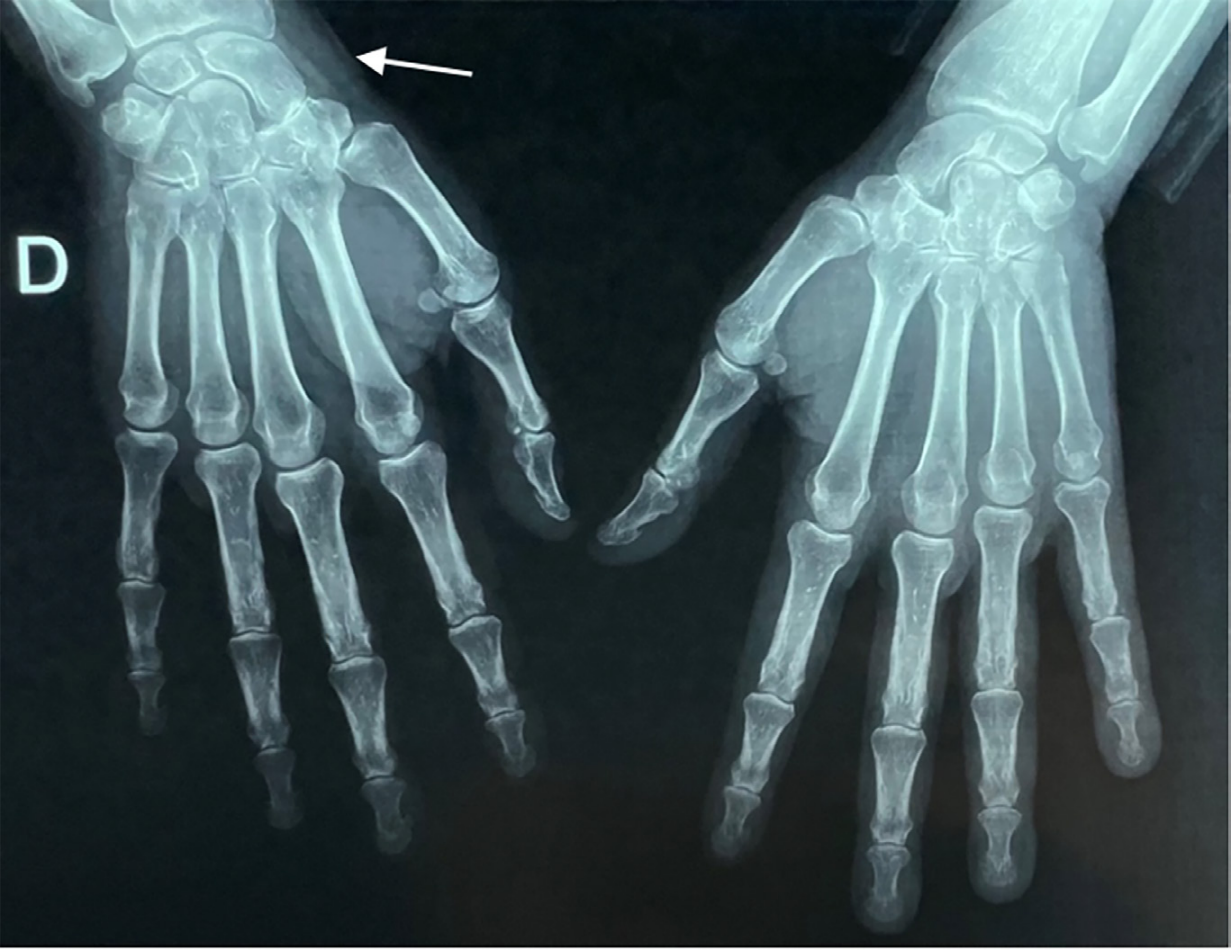
X-ray of the front of the hands and wrists showing thickening of the soft tissues of the right wrist (arrow).

The biological workup revealed a marked inflammatory syndrome: hypochromic microcytic anaemia of inflammatory origin (haemoglobin: 9.9 g dl^−1^), elevated ferritin level (761 ng ml^−1^), leucocytosis at the upper limit of normal (10,000 cells per cubic millimetre), markedly elevated CRP at 197 mg l^−1^ and an increased erythrocyte sedimentation rate of 90 mm h^−1^ (Table 1). A standard anteroposterior radiograph of the right shoulder showed narrowing of the glenohumeral joint space (Fig. 3). A frontal X-ray of the right wrist and hand revealed soft tissue thickening on the lateral aspect of the right wrist (Fig. 4).

**Table 1.** Results of biological, cytological, molecular and histological examinations

Parameter	CRP	ESR	Synovial fluid appearance	Cytological analysis (synovial fluid)	GeneXpert PCR	Histological analysis (synovial tissue)
Results	197 mg l^−1^	90 mm h^−1^	Purulent, thick	>10,000 cells per cubic millimetre	Positive, no rifampicin mutation	Epithelioid and giant cell granulomas with caseous necrosis

ESR, erythrocyte sedimentation rate.

In our case, the primary diagnosis to consider was infectious arthritis in an immunocompromised patient. However, the main diagnostic challenge was distinguishing between an acute and a chronic infection. The patient’s history and clinical examination suggested an acute infection, but tuberculosis had to be considered given the endemic context. The patient’s chronic joint pain could have obscured the true duration of symptoms, as well as the weight loss.

**Fig. 5.**
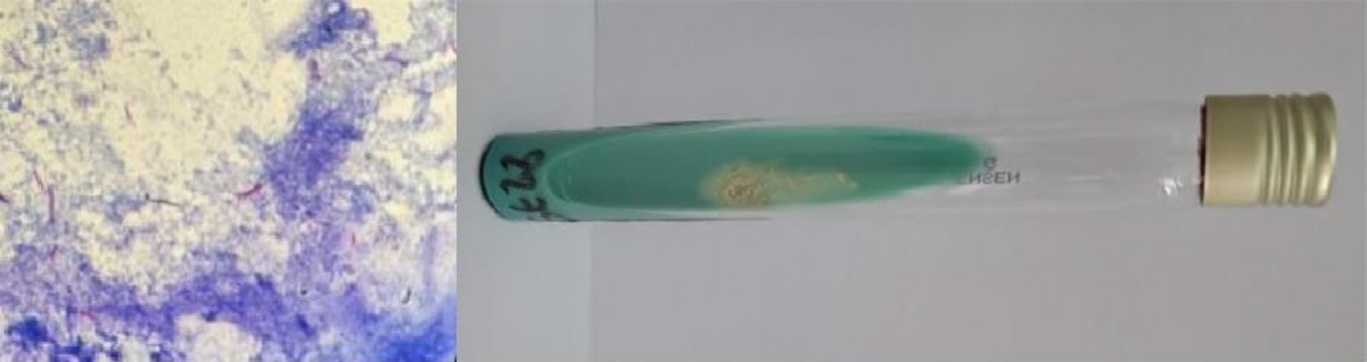
Presence of AFB on Ziehl–Neelsen staining (arrows), with visible colonies in the centre of the Löwenstein–Jensen culture (arrow).

The aspiration of the shoulder joint fluid yielded a thick, purulent material. Cytological analysis revealed a very high cellularity, with a leucocyte count exceeding 10,000 cells per cubic millimetre, predominantly neutrophils, consistent with an inflammatory fluid of septic origin. Direct examination after Ziehl–Neelsen staining showed more than ten acid-fast bacilli (AFB) per field, and culture on Löwenstein–Jensen solid medium became positive after 3 weeks (Fig. 5). Real-time PCR performed using the GeneXpert system (*M. tuberculosis*/rifampicin) confirmed the presence of the *M. tuberculosis* complex, with a high detection threshold and no mutations conferring rifampicin resistance. Moreover, testing for *M. tuberculosis* complex in sputum and urine samples was negative.

Given the acute and highly inflammatory clinical presentation, a superinfection with pyogenic bacteria was suspected, justifying multiplex PCR, joint fluid cultures and blood cultures, all of which were negative.

Initial therapeutic management consisted of surgical lavage of the right glenohumeral joint combined with bursectomy, as well as an intervention on the right wrist. This approach was warranted due to the severity of the clinical presentation, the extent of purulent effusion and the advanced stage of arthritis, clinically confirmed by marked limitation of joint range of motion and significant functional impairment, and radiologically by narrowing of the joint space (Fig. 3). The goal was to provide the patient with the best chance of functional recovery of the shoulder.

Histological analysis performed on synovial tissue fragments collected from the shoulder and wrist during surgery revealed epithelioid and giant cell granulomas containing Langhans giant cells, associated with foci of caseous necrosis, consistent with articular tuberculosis.

The patient was started on a four-drug antituberculous regimen according to the national protocol: isoniazid 300 mg per day, rifampicin 600 mg per day, pyrazinamide 1,500 mg per day and ethambutol 1,200 mg per day for 2 months, followed by dual therapy with isoniazid and rifampicin for 7 months. The case was reported to public health authorities, and the patient’s close contacts were screened. The clinical course was favourable after 2 months of treatment, with marked improvement in the right shoulder and extensor tendons of the right wrist. Functional recovery of joint mobility was achieved through physical rehabilitation sessions.

## Discussion

Osteoarticular tuberculosis is a rare form of extrapulmonary tuberculosis, accounting for 9–20% of cases, with a predilection for the spine, hips and knees [4]. Shoulder and tendon involvement remains exceptional, and diagnosis is frequently delayed due to the lack of specific clinical and radiological features [5]. Several isolated cases have been reported in the literature: Jemaa *et al*. [6] described a case of tuberculous bursitis of the shoulder in a patient with renal failure; Gbané-Koné *et al*. [2] reported a case of shoulder tuberculosis masked by a concomitant *Enterobacter cloacae* infection and Mangwani *et al*. [7] described a pseudoparalytic form mimicking adhesive capsulitis. As for tuberculous tenosynovitis, Walker *et al*. [8] documented a rare form involving the flexor tendons in the context of carpal tunnel syndrome, and Ben Abdelghani *et al*. [9] reported a case affecting the superficial and deep flexor tendons of the second finger of the right hand. Our case is particularly original in that it involves the wrist extensor tendons, an exceptionally rare location, associated with tuberculous monoarthritis of the shoulder.

Systemic diseases are well-recognized risk factors for the reactivation of tuberculosis, especially in immunocompromised settings caused either by the underlying disease or by treatments such as corticosteroids and immunosuppressants. Systemic lupus erythematosus increases this risk by a factor of 5–15 [10]. Tuberculosis should, therefore, be systematically considered in any unusual monoarthritis or oligoarthritis in a patient with systemic disease, particularly in the presence of signs suggestive of tuberculous involvement, such as weight loss, as was the case in our patient.

Systemic scleroderma is a rare autoimmune disease characterized by cutaneous and visceral fibrosis, vascular dysfunction (Raynaud’s phenomenon) and frequent joint pain (arthralgia or arthritis). Respiratory complications include ILD and pulmonary hypertension. Treatment typically involves immunosuppressive therapy, which increases susceptibility to opportunistic infections such as tuberculosis [11].

The onset of acute monoarthritis of the shoulder with extensor tendon tenosynovitis, in this context, was initially mistaken for a flare of systemic scleroderma, which led to delayed consultation and diagnosis. However, the classic presentation of shoulder tuberculosis includes chronic pain and joint stiffness, and sometimes joint locking. The presence of a cold abscess or fistula is rare but highly suggestive [12,13].

Tuberculous tenosynovitis accounts for ~5% of osteoarticular tuberculosis cases, with a predilection for the flexor tendons of the hand and wrist. Involvement of the extensor tendons, as observed in our case, is exceptional and often associated with concomitant joint involvement [14].

Standard radiographs typically reveal Phemister’s triad: periarticular osteoporosis, marginal erosions and initially preserved joint space. In our case, the narrowing of the glenohumeral joint space likely reflects a more chronic evolution, masked by the patient’s scleroderma-related joint pain [4].

Musculoskeletal ultrasound is a valuable tool for detecting joint effusion or destruction, guiding aspiration and potentially avoiding immediate surgical intervention [15]. However, standard biological markers such as erythrocyte sedimentation rate and CRP remain non-specific [16].

Diagnosis relies on the identification of *M. tuberculosis* in synovial fluid: direct examination with Ziehl–Neelsen staining, culture on Löwenstein–Jensen medium (which takes 3–10 weeks) and, more recently, PCR using the GeneXpert system, which is a rapid and specific method [17]. In cases where these techniques fail, synovial biopsy remains essential to demonstrate granulomas with caseous necrosis [18].

The distinctive feature of our case lies in the unusually high concentration of AFB in the joint fluid (shoulder and wrist), likely related to both immunosuppression and diagnostic delay. The tuberculous origin of the tenosynovitis was strongly suspected due to its clinical evolution parallel to that of the shoulder and was confirmed by microbiological tests, Ziehl–Neelsen staining, culture and GeneXpert PCR, performed on the aspirated fluid.

Prolonged treatment with antituberculosis drugs, extending over several months, remains the cornerstone of therapy, although the optimum duration is still under discussion [19]. According to Morocco’s National Tuberculosis Control Program, the treatment regimen for osteoarticular tuberculosis comprises two phases: an initial phase involving a combination of four antituberculosis drugs (rifampicin, isoniazid, pyrazinamide and ethambutol) for 2 months and a secondary phase involving a combination of two antituberculosis drugs (isoniazid and rifampicin) for 7 months [1, 13]. However, in the case of extensive advanced lesions or an inadequate response to chemotherapy, surgery such as debridement, synovectomy or curettage is often necessary [2,19].

In our case, the management was both medical and surgical due to the severity and progressive nature of the lesions (presence of purulent joint fluid affecting the bursa and joint space narrowing). The objective was to optimize the patient’s chances of regaining shoulder function.

## Abbreviations

BCG: Bacillus Calmette Guerin
CRP: C-reactive protein
CT: computed tomography
ESR: erythrocyte sedimentation rate
ILD: interstitial lung disease
NYHA: New York Heart Association
